# Comment to: Post-vaccination incidence and side effects of COVID-19 in a cohort of Brazilian healthcare professionals: an internet-based survey

**DOI:** 10.31744/einstein_journal/2023CE0403

**Published:** 2023-03-24

**Authors:** Rujittika Mungmunpuntipantip, Viroj Wiwanitkit

**Affiliations:** 1 Private Academic Consultant Bangkok Thailand Private Academic Consultant, Bangkok, Thailand.; 2 Joseph Ayo Babalola University Osun State Nigeria Joseph Ayo Babalola University, Ikeji-Arakeji, Osun State, Nigeria;; Dr. D.Y. Patil Medical College Kolhapur India Dr. D.Y. Patil Medical College, Kolhapur, India;; Dr. D. Y. Patil Vidyapeeth Pune India Dr. D. Y. Patil Vidyapeeth, Pune, India.

Dear Editor,

We would like to share our feedback on the publication “Post-vaccination incidence and side effects of COVID-19 in a cohort of Brazilian healthcare professionals: an internet-based survey”.^( 1 )^ Ballestero et al. looked into the prevalence of vaccine adverse effects and COVID-19 among healthcare workers who had been vaccinated.^( 1 )^According to Ballestero et al., 60% of Brazilian healthcare workers had side effects, with local swelling/pain, fatigue/weariness, fever, headache, and limb pain being the most prevalent ones.^( 1 )^ It is still challenging to identify the precise clinical association because limited information on the health and immunological status of vaccination recipients prior to inoculation is available. If people are given misleading information, they may react negatively to immunizations and lose faith in them. The patient’s comorbidities may be the cause of the problem and may lead to misunderstanding.^( 2 )^ In addition to their obvious clinical condition, the vaccine recipients may also have a multitude of co-occurring diseases. Before making any inferences about the presence of an adverse event connected to a vaccine, any further aggravating variables must be ruled out. Therefore, future research and careful planning are required. Studies have shown that inherited genetic variation affects how vaccine recipients’ immune systems react. Inherited genetic variation has been looked into and proven to influence vaccine recipients’ immunological responses.^( 3 )^ A test must be performed to rule out any further exacerbating factors, such as comorbidity or an underlying medical condition. Investigations must be performed to exclude any further exacerbating factors, such as comorbidities. It is important to evaluate the COVID-19 vaccine’s efficacy in a group of people with documented immunological histories and health issues.^( 4 )^
